# Chitosan-Functionalized Graphene Oxide as a Potential Immunoadjuvant

**DOI:** 10.3390/nano7030059

**Published:** 2017-03-08

**Authors:** Ting Yan, Huijie Zhang, Dandi Huang, Shini Feng, Morihisa Fujita, Xiao-Dong Gao

**Affiliations:** Key Laboratory of Carbohydrate Chemistry and Biotechnology, Ministry of Education, School of Biotechnology, Jiangnan University, Wuxi 214122, China; dating129@163.com (T.Y.); qwerdandan@163.com (D.H.); fengshini1990@163.com (S.F.); fujita@jiangnan.edu.cn (M.F.)

**Keywords:** graphene oxide, chitosan, protein adsorption, cytokines, adjuvant

## Abstract

The application of graphene oxide (GO) as a potential vaccine adjuvant has recently attracted considerable attention. However, appropriate surface functionalization of GO is crucial to improve its biocompatibility and enhance its adjuvant activity. In this study, we developed a simple method to prepare chitosan (CS)-functionalized GO (GO-CS) and further investigated its potential as a nanoadjuvant. Compared with GO, GO-CS possessed considerably smaller size, positive surface charge, and better thermal stability. The functionalization of GO with CS was effective in decreasing the non-specific protein adsorption and improving its biocompatibility. Furthermore, GO-CS significantly activated RAW264.7 cells and stimulated more cytokines for mediating cellular immune response, which was mainly due to the synergistic immunostimulatory effect of both GO and CS. GO-CS exhibits strong potential as a safe nanoadjuvant for vaccines and immunotherapy.

## 1. Introduction

As a 2D carbon nanosheet that is chemically exfoliated from oxidized graphite, graphene oxide (GO) exhibits numerous fascinating physicochemical properties, such as abundant functional groups, good chemical and thermal stability, and special electrical and mechanical properties [1,2]. Graphene oxide (GO) has recently emerged as a promising material for various applications, including electronic devices, catalysis, and nanocomposite materials [3,4,5,6]. The past few years have witnessed great research achievements using GO in biomedical fields owing to its excellent aqueous processability, large specific surface areas, and surface functionalizability [7,8,9,10]. For example, GO is widely used in biosensing, cellular imaging, cancer therapy, tissue engineering, and drug/gene delivery [11,12,13,14,15,16,17,18,19]. In vivo studies suggest that PEGylated GO could be gradually cleared from the mouse body possibly through renal and fecal excretion [20,21]. Therefore, GO is considered to be promising for biomedical applications. Researchers have recently started to investigate the interaction of GO with the immune system. Several studies revealed that GO could activate immune cells and trigger multiple toll-like receptor-mediated immune responses [22,23,24]. The immune enhancement effect of GO has also been reported, which enables it as a promising immunoadjuvant or carrier for vaccines [25,26,27]. However, numerous challenges exist before further pre-clinical and clinical studies of GO are conducted. Among these challenges, the toxicity of GO has become a substantial concern. Reportedly, GO exhibits cytotoxic effects both in vitro and in vivo, including the overproduction of intracellular reactive oxygen species (ROS), inducing cell apoptosis and causing severe pulmonary inflammation and the formation of granuloma [28,29,30]. Moreover, the adjuvant activity of GO should be optimized to elicit a robust immune response. Therefore, an alternative GO-based adjuvant with excellent biocompatibility and enhanced adjuvant activity should be developed. Numerous in vitro and in vivo studies reported that the toxicity of GO is closely related to its surface functionalization. GO functionalized with various polymers such as PEG, PEI, and dextran reportedly possess good biocompatibility, as well as aqueous dispersibility and stability [29,31,32]. Although considerable achievements have been gained in this field, efforts are still needed to develop simple methods for constructing safe and efficient GO-based adjuvants. 

Chitosan (CS) is a natural cationic polysaccharide that is generally obtained by the deacetylation of chitin. Owing to its beneficial properties, such as good biocompatibility, biodegradability, and low immunogenicity, CS has been studied for various biomedical applications [33,34]. CS has been widely used for the functionalization of nanocarriers to reduce toxicity and improve delivery efficiency [35,36,37]. Furthermore, CS shows an immunostimulatory effect and could serve as a Toll-like receptor 2 (TLR 2) ligand and stimulate strong immune responses [38]. Reportedly, the activation of multiple TLRs would enhance the activity of adjuvants to induce robust immune responses [25,39,40]. GO and CS could activate multiple TLRs. Thus, CS-functionalized GO may exert a superior immune enhancement effect and offer strong potential as an immunoadjuvant.

In the present study, we used CS to functionalize GO and prepared GO-CS nanocomposites, which were synthesized through a simple noncovalent self-assembly method. Then, we studied the protein adsorption behavior on GO-CS and its response to RAW264.7 cells. Functionalization of GO with CS significantly decreases the non-specific protein adsorption on GO. Furthermore, the GO-CS nanocomposites activated RAW264.7 cells and induced more cytokines compared with GO. Taken together, GO-CS could be a promising nanoadjuvant for vaccines or immunotherapy.

## 2. Results and Discussion

### 2.1 Characterization of GO-CS

**Figure 1:**
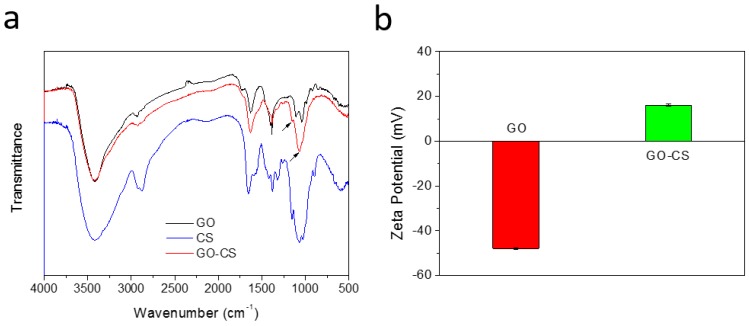
Characterization of graphene oxide (GO) and chitosan (CS) functionalized GO (GO-CS). (**a**) Fourier transform infrared spectroscopy (FTIR) spectra of GO, CS, and GO-CS; (**b**) Zeta potentials of GO and GO-CS.

GO-CS was fabricated through the self-assembly of both GO and CS in aqueous solution via electrostatic interactions. The successful synthesis of the nanocomposite was first validated by Fourier transform infrared spectroscopy (FTIR) (Figure 1). The GO spectrum exhibited two adsorption peaks at 1728 and 1625 cm^−1^, which were assigned to the C=O stretches of the carboxylic group and the deformations of the O–H bond in water, respectively [41]. The peak appearing at 3440 cm^−1^ was ascribed to the hydroxyl group on GO [42]. Two distinct peaks appeared at 1030 and 1593 cm^−1^ in the CS spectrum, which were characteristic of the C=O stretching vibration of -NHCO- and the N-H bending of -NH_2_, respectively [43]. The GO-CS spectrum clearly shows peaks corresponding to both GO and CS, indicating that CS has been functionalized on the GO surface. Furthermore, a peak at 1596 cm^−1^, which was related to the -NH_2_ vibration, disappeared in the GO-CS spectra. Strong electrostatic interactions occurred between the cationic CS and the negatively charged GO. 

**Figure 2:**
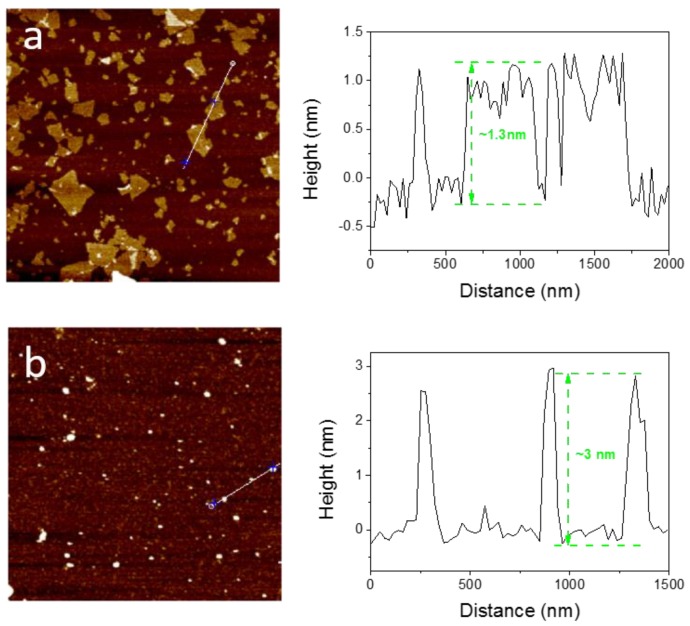
Representative atomic force microscopy images and corresponding height profiles of (**a**) GO and (**b**) GO-CS.

**Figure 3:**
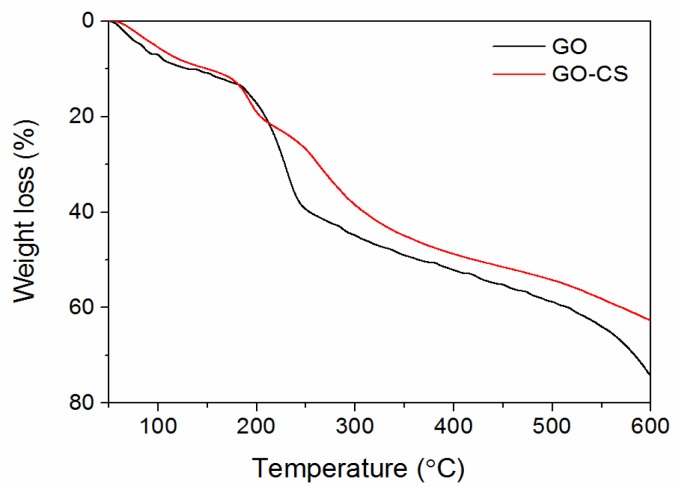
Thermogravimetric analysis curves of GO and GO-CS.

Zeta potential analysis further verified the successful synthesis of GO-CS. The zeta potential of GO was −44 mV. However, after functionalization with cationic CS, GO-CS was positively charged with a zeta potential of +18 mV. Positively charged GO-CS may strongly interact with negatively charged cell membranes and result in enhanced cellular uptake. Moreover, the morphologies of GO and GO-CS were characterized by Atomic force microscopy (AFM). As shown in Figure 2, GO possesses a thickness of approximately 1.3 nm and a lateral size of about several hundred nm (Figure 2a). However, the lateral size of GO-CS was markedly smaller than GO, which was contributed by the sonication step that cut the graphene sheets (Figure 2b). The thickness increased markedly to approximately 3 nm (Figure 2b). A similar increase in sheet thickness was observed when GO was functionalized with PEI [32]. CS was successfully functionalized onto the GO surface, changing its apparent structure and forming the multilayered GO-CS nanocomposite. Furthermore, GO-CS may possess a larger specific surface area because the decrease in size always results in an increase in specific surface area. Thermogravimetric analysis (TGA) is a convenient technique used to reveal the composition and change in the thermal stability of complexes. As shown in Figure 3, GO-CS shows a 63% weight loss in a nitrogen atmosphere at 600 °C, whereas GO exhibits a weight loss of 74%. The grafted CS chains seemed to enhance the thermal stability of GO. The content of CS in GO-CS was about 13%, which was determined by calculating the weight change before and after the functionalization of CS on GO. Therefore, the large surface area and positive charges in GO-CS may improve its antigen loading capacity and delivery efficiency.

### 2.2 CS Functionalization Decreased Non-Specific Protein Adsorption on GO-CS

**Figure 4:**
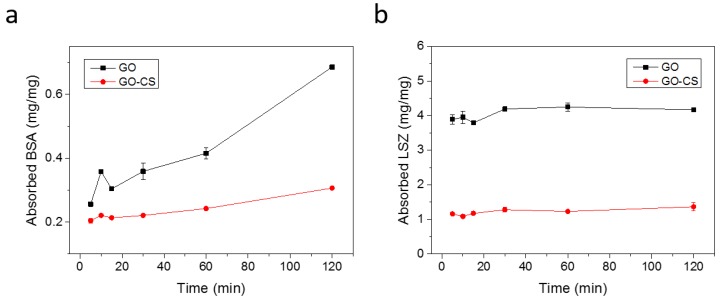
Proteins adsorption on GO and GO-CS. Adsorption of bovine serum albumin (BSA) (**a**) and lysozyme (LSZ) (**b**) on GO and GO-CS versus time.

**Figure 5:**
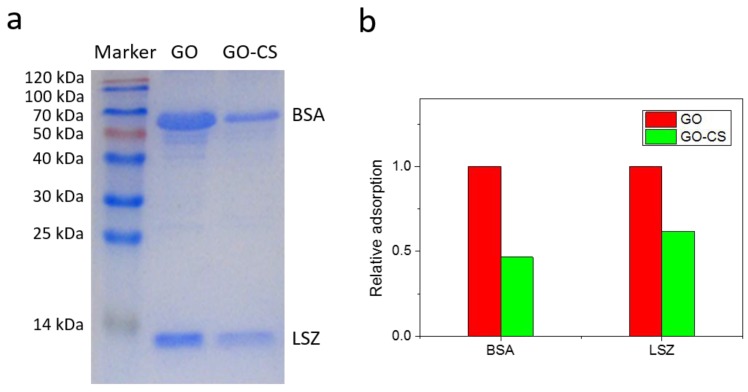
Protein adsorption on GO and GO-CS from a binary mixture of BSA and LSZ. (**a**) Sodium dodecyl sulfate-polyacrylamide gel electrophoresis (SDS-PAGE) of BSA and LSZ adsorbed by GO and GO-CS; (**b**) The calculated relative adsorption based on the corresponding optical density of the bands.

A good drug delivery carrier should possess low non-specific protein adsorption characteristics, which are crucial for limiting the uptake of phagocytic cells and the following immune system clearance. To investigate the protein adsorption on GO and GO-CS, bovine serum albumin (BSA) and lysozyme (LSZ) were selected as model proteins. The amount of BSA and LSZ absorbed on GO and GO-CS is shown in Figure 4. GO shows strong adsorption of both proteins. BSA adsorption occurred in a time-dependent manner, increasing with the time extension. In contrast, the adsorption of LSZ on both materials was a fairly rapid process. LSZ possesses an isoelectric point of 11.1, and thus, LSZ shows a positive zeta potential in PBS. Given that GO possesses a negative zeta potential of about −44 mV, LSZ was expected to strongly interact with negatively charged GO through electrostatic interaction and rapidly bind to GO. Surprisingly, compared with GO, the adsorption of BSA and LSZ significantly decreases after the functionalization of GO with CS. This result is consistent with the previous report that modification of GO with CS/Dextran layers obviously inhibited the BSA adsorption [42]. We further studied the adsorption behavior of GO and GO-CS in the binary mixture of BSA and LSZ, which was characterized by sodium dodecyl sulfate-polyacrylamide gel electrophoresis (SDS-PAGE). As shown in Figure 5a, the proteins bands of BSA and LSZ absorbed on GO-CS were lighter than that of GO, indicating that GO-CS adsorbs a lower amount of BSA and LSZ than GO. This result was further confirmed by analyzing the optical density of the bands on gel (Figure 5b). 

**Figure 6:**
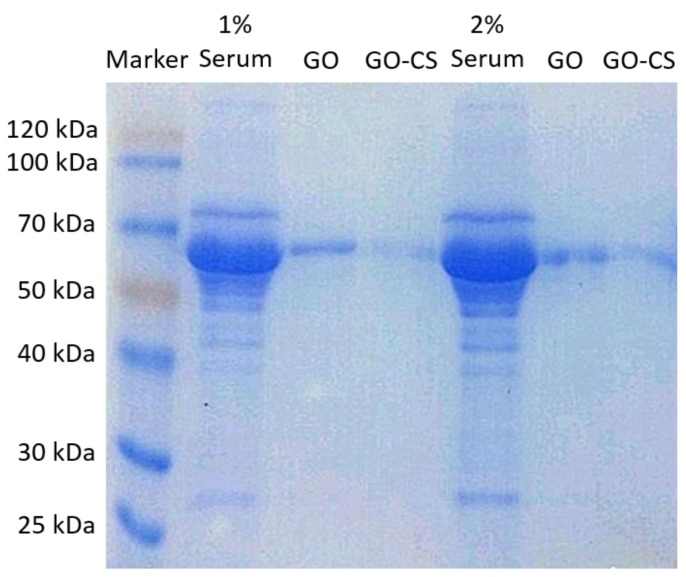
SDS-PAGE of serum protein adsorbed by GO and GO-CS.

Furthermore, when GO and GO-CS were mixed with fetal bovine serum (FBS), which contained a variety of proteins, the amount of proteins adsorbed on GO-CS was considerably lower than that of GO (Figure 6). In addition, both GO and GO-CS adsorbed only a few kinds of proteins. A hydrophobic surface usually causes strong non-specific protein adsorption, whereas materials with a hydrophilic surface show a considerably lower protein adsorption level [42]. Polyethylene glycol (PEG) is widely used as a coating material to decrease non-specific protein adsorption, because PEG increases the hydrophilicity of a material’s surface. Therefore, the functionalization of GO with CS enhances the hydrophilicity of the GO surface and thus decreases the non-specific protein adsorption. Taken together, the functionalization of GO with CS significantly reduces the non-specific protein adsorption, which is beneficial for its biomedical applications.

### 2.3 GO-CS Has Better Biocompatibility

**Figure 7:**
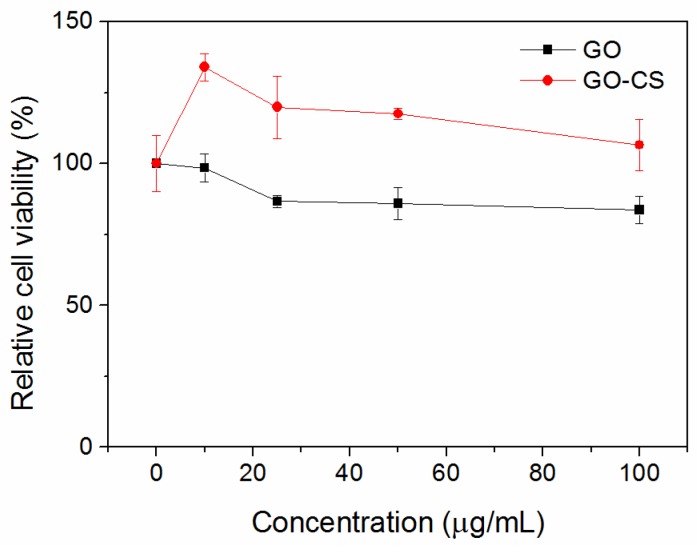
In vitro cytotoxicity assay. Relative cell viability of RAW264.7 cells exposed to indicated concentrations of GO or GO-CS for 24 h was measured by the Cell Counting Kit-8 (CCK-8) assay. Data are presented as mean ± SD (*n* = 5).

Ideal nanomaterials for biomedical applications must possess good biocompatibility. Thus, we evaluated the potential cytotoxicity of GO and GO-CS to RAW264.7 cells by CCK-8 assay. As shown in Figure 7, GO shows evident cytotoxicity to RAW264.7 cells and significantly inhibits their growth, as is consistent with the previous report [23,27,32]. Qu et al. reported that GO induced cell death to macrophages, which was mediated by the activation of toll-like receptor 4 (TLR4) signaling [24]. Additionally, the intracellular accumulation of GO causes cytoskeletal damage and an increase in intracellular ROS, which also decreases the viability and function of macrophages [24]. In contrast, GO-CS exhibits no cytotoxicity and could promote the proliferation of RAW264.7 cells. This result may be attributed to the biocompatibility and accelerating cell proliferation nature of CS in the GO-CS. Moreover, GO with smaller size showed excellent biocompatibility and higher cellular uptake compared to the larger GO nanosheets [44]. Therefore, our data confirms that proper surface coating could efficiently improve the biocompatibility of GO. GO-CS is safe for biomedical applications. 

### 2.4 GO-CS Shows Strong Potential as an Adjuvant

**Figure 8:**
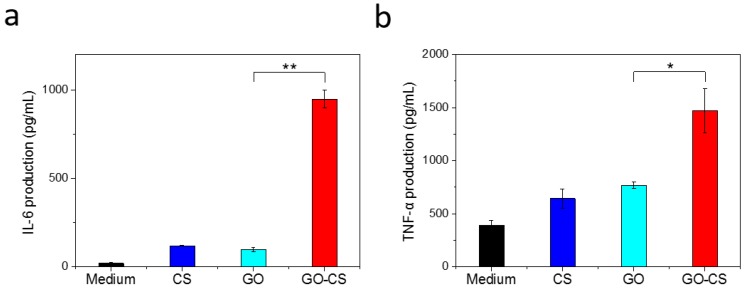
Immunostimulatory activity of CS, GO, and GO-CS. RAW264.7 cells were treated with the indicated materials. The concentration of GO and GO-CS was 50 μg/mL. (**a**) IL-6 production; (**b**) TNF-α production. Data are presented as mean ± SD (*n* = 3). *P < 0.05, **P < 0.01.

As one kind of antigen presenting cells, macrophages perform important roles in the initiation and regulation of immune responses [25]. Upon stimulation by exogenous or endogenous factors, macrophages can release a variety of cytokines and chemokines, which are important for the generation of cellular immunity [23]. To investigate the immunomodulation effect of CS, GO, and GO-CS, we incubated CS, GO, and GO-CS with macrophage-like RAW264.7 cells and measured the cytokine production by using ELISA. As shown in Figure 8, GO induces the secretion of IL-6 and TNF-α to a certain extent from RAW264.7 cells. This result was consistent with the previous report stating that the graphene treatment of macrophages could trigger Toll-like receptor (TLR)-regulated pro-inflammatory responses, which mainly occur through a NF-κB signaling pathway [23]. Tao et al. reported that polyethylene glycol (PEG) and polyethylenimine (PEI) dual-polymer-functionalized GO (GO-PEG-PEI) also induced the TNF-α and IL-6 production to some extent from RAW264.7 cells [27]. CS is widely used as an effective immunoadjuvant, which functions as a TLR2 ligand and induces cytokine secretion [38]. CS induced both IL-6 and TNF-α from RAW 264.7 cells (Figure 8). However, GO-CS induces a markedly higher amount of the IL-6 and TNF-α than GO. Enhanced cytokine production is possibly due to CS functionalization. Given that both GO and CS could activate immune cells, their synergistic effect may result in enhanced cytokine secretion. The smaller size and positive charge that GO-CS possess may enhance its uptake by RAW264.7 cells and further contribute to higher cytokine induction. Taken together, our results suggest that GO-CS could strongly activate RAW264.7 cells and promote the secretion of cytokines, such as IL-6 and TNF-α, which play an important role in activating cellular immunity. Therefore, GO-CS exhibits strong potential as a nanoadjuvant for immunotherapy. Further studies are underway to confirm its adjuvant activity.

## 3. Materials and Methods 

### 3.1. Materials

GO solution (2 mg/mL) was obtained from XFNANO Nanomaterials Tech Co., Ltd. (Nanjing, China). Water soluble chitosan with a molecular weight of 60–120 kDa and lysozyme (LYZ) from chicken egg were purchased from Sigma-Aldrich (Saint Louis, MO, USA). Bovine serum albumin (BSA) was purchased from Biosharp (Shanghai, China). The BCA Protein Assay Kit was obtained from Beyotime (Shanghai, China). Dulbecco’s Modified Eagle’s Medium (DMEM) and fetal bovine serum (FBS) were obtained from Gibco (Grand Island, NY, USA). The mouse leukemic monocyte macrophage RAW264.7 cell line was purchased from the Cell Bank of Chinese Academy of Sciences (Shanghai, China). Cell Counting Kit-8 (CCK-8) was purchased from Dojindo (Kumamoto, Japan). All chemicals were used as received.

### 3.2. Preparation of GO-CS 

CS solution (1 wt %) was prepared by dissolving CS in 1% (*v/v*) acetic acid solution. In a typical procedure, 0.5 mL of GO solution (0.5 mg/mL) was firstly ultrasonicated for 10 min, and then, an equal volume of CS solution was gradually added to the GO solution with stirring, followed by ultrasonication for another 20 min. After stirring for 2 h at room temperature, the GO-CS was collected by centrifugation and washed 3 times with Milli-Q water. The obtained GO-CS was re-dispersed in PBS.

### 3.3. Characterization

Atomic force microscopy (AFM) images were obtained using tapping mode by a Dimension Icon atomic force microscopy (Bruker, Billerica, MA, USA). Fourier transform infrared (FTIR) spectra were recorded on a Nexus spectrophotometer (Nicolet, Madison, WI, USA) at 4 cm^−1^ resolution with 32 scans. Zeta potential analyses were performed using a Zetasizer Nano ZS instrument (Malvern, UK). Thermogravimetric analysis (TGA) was conducted on TGA/SDTA851e system (Mettler Toledo, Switzerland) at a heating rate of 5 °C per minute under a nitrogen atmosphere.

### 3.4. Protein Adsorption

BSA, LSZ, and FBS were selected as model proteins to evaluate the protein adsorption on GO and GO-CS. Protein adsorption experiments were carried out at room temperature. 250 µL of GO and GO-CS (0.2 mg/mL) were mixed with equal volumes of BSA or LSZ with a concentration of 500 µg/mL. After incubation with stirring for an indicated time, the supernatant was removed by centrifugation and the precipitate was obtained. The precipitate was washed with PBS twice and then resuspended in 2% SDS, followed by sonication for 30 s and stirring for 1 h to desorb the proteins from the nanomaterials. The amount of adsorbed proteins were determined by the BCA Protein Assay Kit. The absorbance at 562 nm was measured by an Enspire 2300 microplate reader ((Waltham, MA, USA). To investigate the effect of pH on protein adsorption, protein adsorption experiments were conducted in PBS with different pH values, and an incubation time of 2 h was adopted.

In binary protein or multiple protein systems, protein adsorption experiments were carried out using the same procedures as described above. Binary mixtures contained BSA/LSZ with the same concentration of 500 µg/mL. FBS was diluted to 1% (*v/v*) and 2% (*v/v*) and was used in multiple protein adsorption experiments. Protein adsorption on GO and GO-CS was characterized by SDS-PAGE. 10 µL of each sample was loaded in each well and the gels were run for 35 min at 120 V and then stained with Coomassie Brilliant Blue R-250 (Bio-Rad, Hercules, CA, USA). The optical density of the bands was analyzed using the Image J software.

### 3.5. Cell Culture and Proliferation Assay 

Mouse macrophage cell line RAW264.7 cells were maintained in DMEM medium supplemented with 100 U/mL penicillin, 100 mg/mL streptomycin, and 10% heat-inactivated FBS at 37 °C with a humidified atmosphere containing 5% CO_2_. The influence of GO and GO-CS exposure on the proliferation of RAW264.7 cells was determined by CCK-8 assay. Cells were seeded in 96-well plates and incubated overnight to allow the cells to adhere. Then, the cells were exposed to GO and GO-CS with increasing concentrations (10, 25, 50, and 100 µg/mL, respectively). After incubation at 37 °C for 24 h, the CCK-8 solution was added to each well. Then the cells were incubated for another 3 h in the CO_2_ incubator. The absorbance of each well was measured at 450 nm by a Bio-Rad 680 microplate reader (Hercules, CA, USA). Relative cell viability was calculated as the fraction of treated cells/untreated cells.

### 3.6. Cytokine Assay 

RAW264.7 cells were seeded in a 96-well plate at a density of 1×10^5^ cells/well. After 24 h of incubation, cells were treated with CS, GO (50 µg/mL), and GO-CS (50 µg/mL) at 37 °C for another 24 h (IL-6) or 8 h (TNF-α). Then the supernatant of the cell culture was collected and the secreted IL-6 and TNF-α were measured by ELISA using the Ready-SET-Go! Mouse IL-6 kit (eBioscience, San Diego, CA, USA) and Mouse TNF-α kit (eBioscience, San Diego, CA, USA) according to the manufacturer’s instructions.

### 3.7. Statistical Analysis

Statistical analysis was performed using Student’s *t*-test. Data are presented as mean ± standard deviation. Differences were considered to be statistically significant (* *p* < 0.05, ** ** < 0.01).

## 4. Conclusions

In this study, we successfully prepared the GO-CS nanocomposite through a simple noncovalent self-assembly method. GO-CS presents a smaller size and positive surface charge. Functionalization of GO with CS not only decreased the non-specific protein adsorption, but also improved its biocompatibility. The current work indicates that GO-CS strongly activated marcophages and stimulated more cytokines involved in cellular immune responses, suggesting GO-CS to be a good adjuvant candidate for vaccines and immunotherapy.
